# Optimizing venetoclax duration with hypomethylating agents for newly diagnosed acute myeloid leukemia: impact on response and survival

**DOI:** 10.1007/s00277-026-07219-2

**Published:** 2026-08-04

**Authors:** Anush A. Ginosyan, Karam Ashouri, Justin Cheng, Lauren Ford, Mihir Baya, Lucas Humayun, Danielle Hong, Elaine Huang, Grace Kim, Brandon Tang, Brian Hom, Robert Ireland, Imran Siddiqi, Amir Ali, Karrune Woan, Eric Leon Tam, George Yaghmour

**Affiliations:** 1 Keck School of Medicine, University of Southern California, Los Angeles, CA USA; 2 Department of Pathology, Norris Comprehensive Cancer Center, University of Southern California, Los Angeles, CA USA; 3 Department of Pharmacy, Norris Comprehensive Cancer Center, University of Southern California (USC), Los Angeles, CA USA; 4 Jane Anne Nohl Division of Hematology and Center for the study of Blood disease, Division of Hematology, Norris Comprehensive Cancer Center, University of Southern California, Los Angeles, CA USA

**Keywords:** AML, Venetoclax, Hypomethylating agents, Treatment duration, Elderly, Survival outcomes

## Abstract

The addition of venetoclax to hypomethylating agents (HMAs) is standard for older adults with newly diagnosed acute myeloid leukemia (AML). However, prolonged venetoclax (VEN) exposure can cause cytopenias and infections, raising questions about optimal duration. We conducted a single‑center retrospective study of 102 patients treated between 2018 and 2025 with azacitidine or decitabine plus VEN administered for 14 (*n* = 22), 21 (*n* = 48), or 28 (*n* = 32) days per 28‑day cycle. Baseline cytogenetic and molecular profiling, responses by 2022/2024 ELN criteria, measurable residual disease (MRD), cumulative incidence of relapse (CIR), disease‑free survival (DFS), and overall survival (OS) were analyzed using competing‑risks regression and Cox models. Median age was 68 years and patients received a median of 4 cycles. Composite remission (CR/CRi/CRh/MLFS) was 66.7%, with MRD negativity in 75.0% of responders. Remission and MRD negativity rates did not differ significantly across VEN‑duration cohorts. Median OS and DFS were 17.3 and 12.3 months, respectively, with no significant differences in OS, DFS, and CIR between 14‑, 21‑, and 28‑day cohorts. Grade ≥ 3 cytopenias and transfusion independence rates also did not differ significantly. In this cohort, shortening VEN exposure to 14 or 21 days did not show a significant difference in response, survival, relapse risk, and toxicity profiles relative to a 28‑day schedule in our cohort. These findings support further prospective evaluation of reduced VEN durations in this population.

**Supplementary Information:**

The online version contains supplementary material available at https://doi.org/10.1007/s00277-026-07219-2.

## Introduction

The management of elderly patients with acute myeloid leukemia (AML) presents significant challenges due to age-related factors and comorbidities that may limit their eligibility for intensive chemotherapy regimens or allogeneic transplant. Historically, less intensive approaches such as low‑dose cytarabine or azacitidine (AZA) monotherapy have been employed in this patient population, with suboptimal outcomes and complete remission (CR) rates below 30% for azacitidine monotherapy [1]. The introduction of venetoclax (VEN), a BCL2 inhibitor, in combination with a hypomethylating agent (HMA), has emerged as the standard of care for older adults with newly diagnosed AML following the success of the phase 3 VIALE‑A trial [2]. However, despite favorable survival results, high rates of hematologic adverse events were reported in the VEN + AZA group, including increased rates of febrile neutropenia [2].

Several institutions have investigated shortening the duration of venetoclax to reduce prolonged cytopenias and infection risks. While some studies suggest that shorter courses may preserve efficacy [3], the impact of abbreviated venetoclax exposure on long-term disease control and relapse is not well established, particularly in patients with greater mutational burden and unfavorable risk by the 2022/2024 European LeukemiaNet (ELN) criteria.

## Methods

We retrospectively studied consecutive adult patients with newly diagnosed acute myeloid leukemia treated at the University of Southern California (USC) Norris Cancer Center between 2018 and 2025 who received VEN + HMA as induction therapy for 14, 21, or 28 days in a 28‑day cycle, per treating physician discretion. Shortened venetoclax durations were either pre-specified from cycle 1, typically in patients whose comorbidities were expected to limit tolerability, or reduced dynamically during treatment in response to factors such as rapid peripheral blast clearance, infection, or other complications. Specifically, five patients had treatment dynamically changed from 21 day to 14 day, three patients for early clearance of peripheral blasts and two for severe infections. The study was approved by the Institutional Review Board at the University of Southern California and was conducted in accordance with the Declaration of Helsinki.

Venetoclax was administered with a standard intra-cycle ramp-up to a target dose of 400 mg daily. In accordance with the venetoclax prescribing information, the dose was reduced for concomitant CYP3A inhibitors used for antifungal prophylaxis or treatment: by 50% (to 200 mg daily) with moderate inhibitors (e.g., fluconazole, isavuconazole) and by ≥ 75% with strong inhibitors (to 100 mg with voriconazole) [4, 5].

Cytogenetic and molecular studies were performed using conventional karyotyping and next‑generation sequencing, respectively. Genetic risk and treatment response was assessed according to the 2022 and 2024 ELN criteria [6, 7]. Measurable residual disease (MRD) was assessed on bone marrow aspirate by multiparameter flow cytometry using integrated leukemia-associated immunophenotype (LAIP) and different-from-normal (DfN) approaches, with an analytical sensitivity (limit of detection) of 0.01%. In accordance with the 2021 European LeukemiaNet MRD Working Party recommendations, MRD positivity was defined as the presence of ≥ 0.1% of CD45-expressing cells with an aberrant leukemia-associated immunophenotype [8]. MRD was evaluated on the bone marrow obtained at the time of best morphologic response (CR/CRi/CRh/MLFS), during induction [8]. The primary endpoint was overall survival (OS), defined as the time from initiation of VEN + HMA therapy to death from any cause. Secondary endpoints were disease-free survival (DFS; time from achievement of remission to relapse or death from any cause), cumulative incidence of relapse (CIR; time from achievement of remission to relapse, with death as a competing event), the composite complete remission rate (CR/CRi/CRh/MLFS), MRD negativity, and treatment-related toxicity graded according to CTCAE v5.0. OS and DFS were analyzed using Cox proportional hazards models and CIR using competing-risks (Fine-Gray) regression. Given the retrospective, single-center, non-randomized design and the limited sample size within each duration cohort, all analyses were exploratory and hypothesis-generating; no formal sample-size or power calculation was performed, and no adjustment was made for multiple comparisons.

## Results

### Patient population

Our patients (*n* = 102; median age 68 years; 59.8% male; 63.4% de novo) received a median of 4 cycles (range 1–30) of azacitidine (*n* = 34) or decitabine (*n* = 68) with a median venetoclax dose of 200 mg daily (range 100–400 mg) for 14 days (*n* = 22), 21 days (*n* = 48), or 28 days (*n* = 32). Baseline characteristics were similar across groups, though the 14-day group compared to the 21- or 28-day cohorts trended toward a higher prevalence of adverse ELN 2022 classification (90.9% vs. 59.6% vs. 61.3%, *p* = 0.054) and trended toward higher prevalence of secondary AML (14-day vs. 21-day vs. 28-day: 54.5% vs. 25.5% vs. 40.6%, *p* = 0.057) (Table 1). The most common mutations within our cohort were TP53 (27%), DNMT3A (17%), NPM1 (17%), IDH2 (15%), and TET2 (15%) (Figure S1). A separate analysis of baseline clinical factors, ELN risk group, cytogenetics, and mutational status was performed.

**Table 1:** Demographics and clinical characteristics stratified by duration of venetoclax

	14-day (*N* = 22)	21-day (*N* = 48)	28-day (*N* = 32)	Total (*N* = 102)	*p* value
Age	68.0 (27.0, 89.0)	69.0 (31.0, 83.0)	67.0 (35.0, 91.0)	68.0 (27.0, 91.0)	0.6581
Sex					0.8992
Female	8 (36.4%)	19 (39.6%)	14 (43.8%)	41 (40.2%)	
Male	14 (63.6%)	29 (60.4%)	18 (56.2%)	61 (59.8%)	
Race/Ethnicity					0.5782
African American	2 (9.1%)	1 (2.1%)	5 (15.6%)	8 (7.9%)	
Asian	1 (4.5%)	4 (8.5%)	1 (3.1%)	6 (5.9%)	
Caucasian	8 (36.4%)	19 (40.4%)	14 (43.8%)	41 (40.6%)	
Hispanic	8 (36.4%)	14 (29.8%)	8 (25.0%)	30 (29.7%)	
Other	3 (13.6%)	9 (19.1%)	4 (12.5%)	16 (15.8%)	
AML type					0.0572
De Novo	10 (45.5%)	35 (74.5%)	19 (59.4%)	64 (63.4%)	
Secondary	12 (54.5%)	12 (25.5%)	13 (40.6%)	37 (36.6%)	
2022 ELN category					0.0542
Favorable	0 (0.0%)	9 (19.1%)	7 (22.6%)	16 (16.0%)	
Intermediate	2 (9.1%)	10 (21.3%)	5 (16.1%)	17 (17.0%)	
Adverse	20 (90.9%)	28 (59.6%)	19 (61.3%)	67 (67.0%)	
Cytogenetics					0.1332
Abnormal	4 (20.0%)	5 (10.9%)	3 (10.3%)	12 (12.6%)	
Complex	10 (50.0%)	11 (23.9%)	10 (34.5%)	31 (32.6%)	
Monosomal	1 (5.0%)	5 (10.9%)	6 (20.7%)	12 (12.6%)	
Normal	5 (25.0%)	25 (54.3%)	10 (34.5%)	40 (42.1%)	
2024 ELN category					0.6892
Adverse	5 (26.3%)	11 (23.4%)	12 (38.7%)	28 (28.9%)	
Favorable	12 (63.2%)	29 (61.7%)	16 (51.6%)	57 (58.8%)	
Intermediate	2 (10.5%)	7 (14.9%)	3 (9.7%)	12 (12.4%)	
EMD					0.6002
No	20 (90.9%)	44 (91.7%)	27 (84.4%)	91 (89.2%)	
Yes	2 (9.1%)	4 (8.3%)	5 (15.6%)	11 (10.8%)	
Venetoclax cycles	3.0 (1.0, 16.0)	4.0 (1.0, 30.0)	3.5 (1.0, 20.0)	4.0 (1.0, 30.0)	0.3441
Hypomethylating agent					0.4462
Azacitidine	9 (40.9%)	13 (27.1%)	12 (37.5%)	34 (33.3%)	
decitabine	13 (59.1%)	35 (72.9%)	20 (62.5%)	68 (66.7%)	
Cycle 1 dose (mg)	200.0 (100.0, 400.0)	200.0 (100.0, 400.0)	200.0 (100.0, 400.0)	200.0 (100.0, 400.0)	0.0011
Transplant					0.5982
No	18 (81.8%)	36 (75.0%)	27 (84.4%)	81 (79.4%)	
Yes	4 (18.2%)	12 (25.0%)	5 (15.6%)	21 (20.6%)	
Achieved CR/CRi					0.2992
CR/CRi	11 (50.0%)	32 (66.7%)	17 (53.1%)	60 (58.8%)	
No	11 (50.0%)	16 (33.3%)	15 (46.9%)	42 (41.2%)	
Achieve CR/CRi/CRh/MLFS					0.1082
No	9 (40.9%)	11 (22.9%)	14 (43.8%)	34 (33.3%)	
Yes	13 (59.1%)	37 (77.1%)	18 (56.2%)	68 (66.7%)	
MRD at CR					1.0002
MRD negative	10 (76.9%)	28 (75.7%)	13 (72.2%)	51 (75.0%)	
MRD positive	3 (23.1%)	9 (24.3%)	5 (27.8%)	17 (25.0%)	
Thrombocytopenia ≥ Grade 3					0.8302
No	12 (57.1%)	25 (54.3%)	12 (46.2%)	49 (52.7%)	
Yes - Grade 3	0 (0.0%)	2 (4.3%)	2 (7.7%)	4 (4.3%)	
Yes - Grade 4	9 (42.9%)	19 (41.3%)	12 (46.2%)	40 (43.0%)	
Febrile neutropenia					0.8702
No	15 (71.4%)	32 (71.1%)	15 (65.2%)	62 (69.7%)	
Yes	6 (28.6%)	13 (28.9%)	8 (34.8%)	27 (30.3%)	
Neutropenia ≥ Grade 3					0.7892
No	9 (42.9%)	24 (52.2%)	13 (50.0%)	46 (49.5%)	
Yes - Grade 3	2 (9.5%)	5 (10.9%)	1 (3.8%)	8 (8.6%)	
Yes - Grade 4	10 (47.6%)	17 (37.0%)	12 (46.2%)	39 (41.9%)	
TLS					0.2992
No	17 (89.5%)	39 (90.7%)	23 (100.0%)	79 (92.9%)	
Yes	2 (10.5%)	4 (9.3%)	0 (0.0%)	6 (7.1%)	
Platelet transfusion independent					0.8332
No	11 (78.6%)	23 (69.7%)	15 (68.2%)	49 (71.0%)	
Yes	3 (21.4%)	10 (30.3%)	7 (31.8%)	20 (29.0%)	
RBC transfusion independent					0.7042
No	11 (78.6%)	22 (66.7%)	14 (63.6%)	47 (68.1%)	
Yes	3 (21.4%)	11 (33.3%)	8 (36.4%)	22 (31.9%)	
30-Day Mortality					1.0002
No	22 (100.0%)	47 (97.9%)	32 (100.0%)	101 (99.0%)	
Yes	0 (0.0%)	1 (2.1%)	0 (0.0%)	1 (1.0%)	
60-Day Mortality					0.5512
No	21 (95.5%)	47 (97.9%)	30 (93.8%)	98 (96.1%)	
Yes	1 (4.5%)	1 (2.1%)	2 (6.2%)	4 (3.9%)	

*AML* acute myeloid leukemia, *ELN* European leukemia network, *EMD* extramedullary disease, *mg* milligram, *CR* complete remission, *CRi* complete remission with incomplete count recovery, *MRD* measurable residual disease, *TLS* Tumor Lysis Syndrome

1. Wilcoxon-rank sum test

2. Fisher’s Exact Test

### Treatment response

Our cohort had a median follow up of 28.1 months (reverse Kaplan Meier method), 51 deaths (50%) and 21 (20.6%) allogeneic hematopoietic stem cell transplants. CR or CR with incomplete hematologic recovery (CRi) occurred in 60 (58.8%) patients and 8 (7.8%) patients achieved CR with partial hematologic recovery (CRh) or morphologic leukemia-free state (MLFS). Of those who achieved remission (CR/CRi/CRh/MLFS), 51 (75.0%) were measurable residual disease (MRD) negative. CR/CRi and MRD negativity rates were not significantly different across cohorts (50.0% vs. 66.7% vs. 53.1%, *p* = 0.299, and 76.9% vs. 75.7% vs. 72.2%, *p* = 1, respectively). Analysis of CR/Cri rates by venetoclax duration with ELN 2022 and 2024 risk groups were also performed (Table S1).

### Survival outcomes

Median OS and DFS in our cohort were 17.3 and 12.3 months, respectively. Two-year OS, DFS, and CIR were 39.6% (95%CI 29.5–53.2), 36.3% (95%CI 25.4–51.8), and 45.6% (95%CI 32.0-58.2), respectively.

**Fig. 1:**
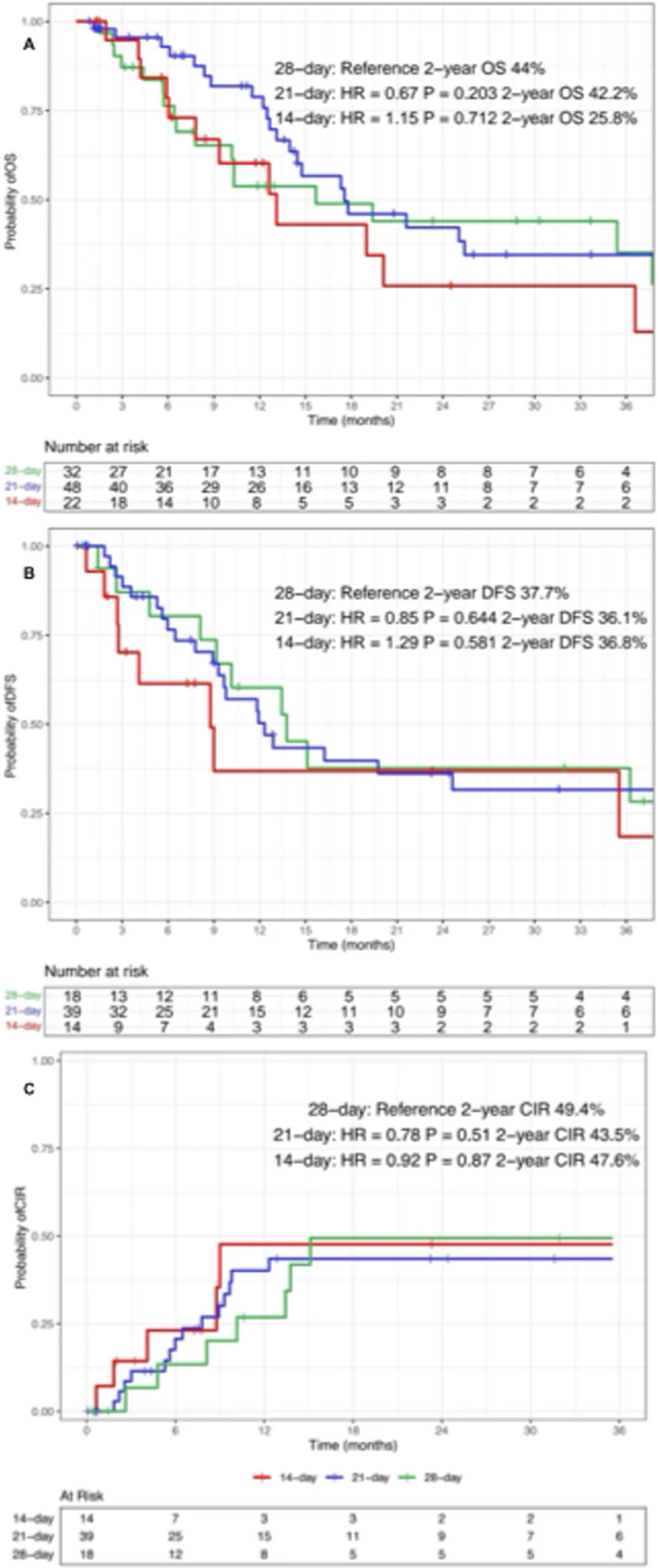
Kaplan Meier Curves by venetoclax duration (**A**) overall survival, (**B**) disease free survival, and (**C**) cumulative incidence of relapse

When compared to the 28-day group, the 14-day group had no significant difference in OS (HR 1.15, 95%CI 0.55–2.37, *p* = 0.712), DFS (HR 1.29, 95%CI 0.52–3.18, *p* = 0.581), and CIR (HR 0.92, 95%CI 0.31–2.70, *p* = 0.87). Similarly, the 21-day group also demonstrated no significant difference in OS (HR 0.67, 95%CI 0.36–1.24, *p* = 0.203), DFS (HR 0.85, 95%CI 0.41–1.72, *p* = 0.644), and CIR (HR 0.78, 95%CI 0.37–1.65, *p* = 0.51). Two-year OS rates were 25.8% (14-day), 42.2% (21-day), and 44.0% (28-day), two-year DFS rates were 36.8%, 36.1%, and 37.7%, respectively, while two-year CIR rates were 47.6%, 43.5%, and 49.4%, respectively. (Fig. 1)

### Univariate and multivariate analysis

**Fig. 2:**
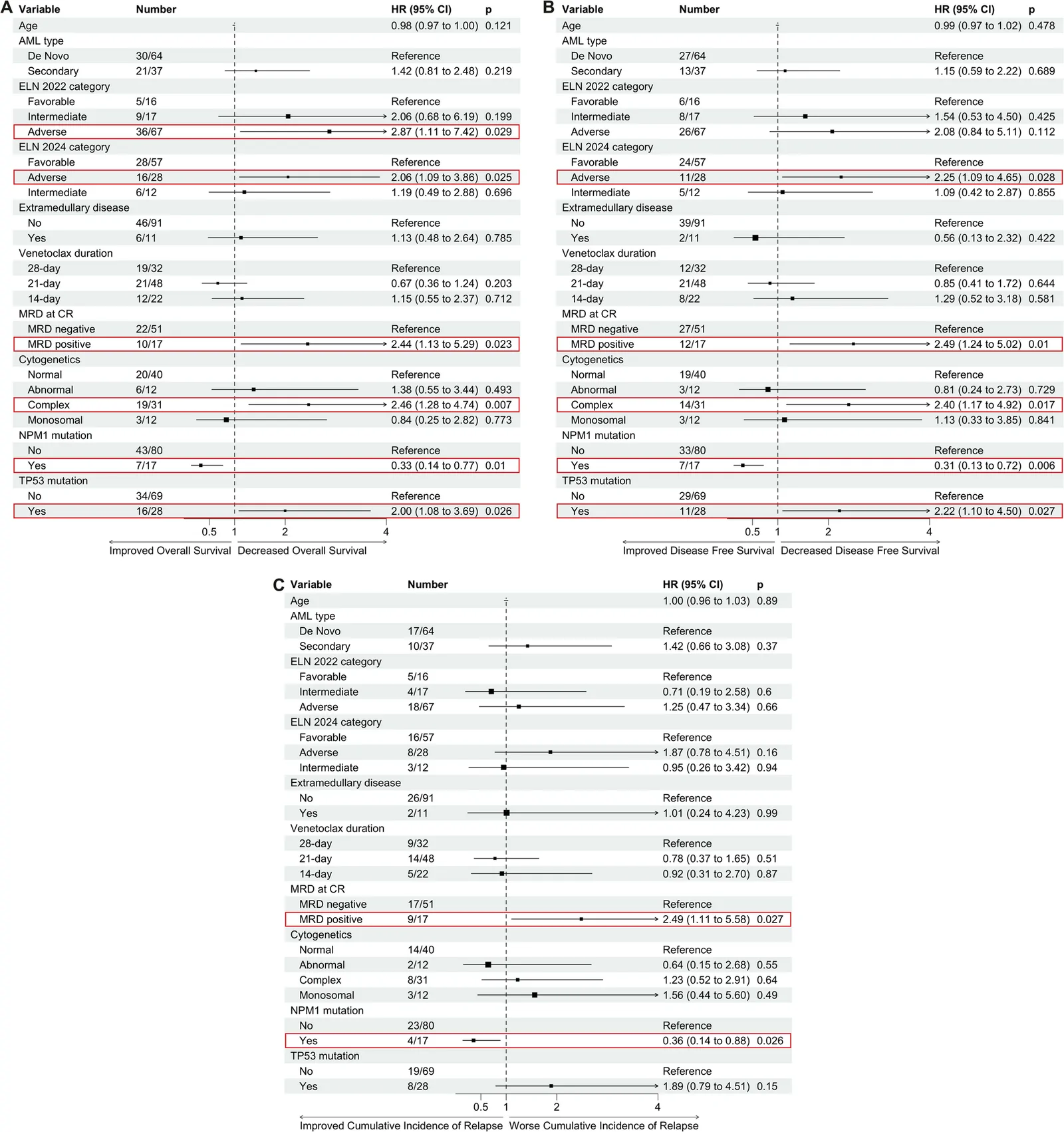
Forest plots of univariate analysis for (**A**) overall survival, (**B**) disease free survival, and (**C**) cumulative incidence of relapse. *Abbreviations*: AML = acute myeloid leukemia; ELN = European leukemia network; MRD = measurable residual disease; CR = complete remission

On univariate analysis, adverse ELN 2022 risk group (HR 2.87, 95% CI 1.11–7.42, *p* = 0.029), adverse ELN 2024 (HR 2.06, 95% CI 1.09–3.86, *p* = 0.025), complex cytogenetics (HR 2.46, 95% CI 1.28–4.74, *p* = 0.007), TP53 mutation (HR 2.00, 95% CI 1.08–3.69, *p* = 0.026) and MRD positivity. at CR (HR 2.44, 95% CI 1.13–5.29, *p* = 0.023) were associated with inferior OS, whereas NPM1 mutation (HR 0.35, 95% CI 0.14–0.77, *p* = 0.01) was associated with improved OS. Predictors of worse DFS were positive MRD at CR (HR 2.49, 95% CI 1.24–5.02, *p* = 0.01), adverse ELN 2024 (HR 2.25, 95% CI 1.09–4.65, *p* = 0.028), TP53 mutation (HR 2.22, 95% CI 1.10–4.50, *p* = 0.027), and complex cytogenetics (HR 2.40, 95% CI 1.17–4.92, *p* = 0.017), while NPM1 mutation (HR 0.31, 95% CI 0.13–0.72, *p* = 0.006) conferred improved DFS. Positive MRD at CR (HR 2.49, 95% CI 1.11–5.58, *p* = 0.027) predicted worse CIR, while NPM1 mutation (HR 0.36, 95% CI 0.14–0.88, *p* = 0.026) was associated with improved CIR. (Fig. 2) Multivariate analysis controlling for Age, AML type, and ELN (2022 and 2024 in separate models) revealed no significant difference in OS, DFS, and CIR by venetoclax duration (Supplementary Table 2). Multivariate models were limited to 10 to 15 outcomes per variable.

### Toxicity

Rates of grade *≥* 3 neutropenia and thrombocytopenia were comparable among the 14-day, 21-day, and 28-day cohorts (57.1% vs. 47.9% vs. 50.0%, *p* = 0.789 and 42.9% vs. 45.6% vs. 53.9%, *p* = 0.830, respectively). Rates of transfusion independence with platelets and RBCs did not differ significantly across cohorts (21.4% vs. 30.3% vs. 31.8%, *p* = 0.833 and 21.4% vs. 33.3% vs. 36.4%, *p* = 0.704, respectively). Rates of tumor lysis syndrome and febrile neutropenia did not differ among cohorts (Table 1). One patient died within 30 days of treatment initiation and four within 60 days (Table 1).

## Discussion

In this cohort, shortening the duration of venetoclax to 14 or 21 was not associated with a statistically significant difference in response rates or survival outcomes compared to the standard 28-day schedule in newly diagnosed AML. However, given wide confidence intervals clinically meaningful differences cannot be excluded. The observed CR/CRi rate of 58.8% and median OS of 17.3 months are comparable with both the pivotal VIALE-A trial results and other real-world datasets [2, 3]. These findings add to growing evidence suggesting that attenuated dosing may achieve clinical parity with continuous exposure [3, 9, 10].

The finding that OS, DFS, and CIR did not significantly differ across the 14, 21, and 28-day cohorts supports recent multicenter retrospective analyses. Karrar et al. (Mayo Clinic) and Kanaya et al. (Hokkaido Leukemia Net) both reported no statistically significant differences in composite remission or overall survival between 14 days and longer venetoclax schedules [3, 9]. This suggests the synergistic induction of leukemia cell death by HMAs and venetoclax occurs early in the treatment cycle, potentially rendering prolonged exposure unnecessary for many patients [3, 11].

Managing myelosuppression is a primary challenge in HMA + VEN therapy. In this study, rates of grade *≥* 3 neutropenia and thrombocytopenia did not differ significantly across the three dosing groups, suggesting that the combination’s inherent toxicity may be triggered during the initial week of treatment regardless of the total duration. This aligns with the Mayo Clinic study, which also found no significant difference in hematological adverse events between 14-day and longer dosing schedules [3].

However, some investigators have observed safety advantages with reduced exposure. Aiba et al. reported a trend toward fewer febrile neutropenia episodes and shorter hospital stays in patients receiving 14 day venetoclax [12]. The optimal extent of shortening nonetheless remains debated. Willekens et al. found that a 7-day “7 + 7” regimen produced comparable response and survival with lower 8-week mortality (6% vs. 16%) [11], whereas the VENAURA analysis by Aspas Requena et al. found 7-day exposure was associated with lower composite remission and inferior survival (HR 2.22) versus 21 days or more, but only in favorable- and intermediate-risk patients, not adverse-risk disease [13]. This mirrors Willekens, whose survival decrement with 7-day venetoclax was likewise limited to the most venetoclax-sensitive patients (NPM1/IDH-enriched, TP53/RAS/FLT3-ITD-negative): in such BCL-2-dependent disease adequate exposure appears necessary, whereas TP53-mutated, adverse-risk AML fares poorly regardless of duration [14]. Similarly, we found increasing durations of venetoclax to be associated with numerically higher CR/CRi rates in subgroups of ELN 2024 favorable (14 day: 42% vs. 21 day: 76% vs. 28 day: 63%) and Intermediate risk (50% vs. 57% vs. 100%). Although it is important to note these p-values were not significant, likely due to small numbers within the subgroup analysis and are exploratory. Early results from the OPTI-AML trial failed to demonstrate non-inferiority for CR rates between 14-day (43%) and 28-day (49%) venetoclax (95% CI difference − 8% to 21% exceeding > 10% threshold) [15]. However, patients with NPM1 and IDH mutations appear to have higher CR rates with longer duration venetoclax (61% vs. 42%).

Molecular and cytogenetic risk factors remain the primary determinants of outcome. Consistent with broader clinical data, NPM1 mutations were associated with significantly improved OS and DFS, while TP53 mutations and adverse ELN risk (both 2022 and 2024) predicted inferior survival [6]. These results are reinforced by Stengel et al., who identified TP53 double-hit alterations as the strongest negative prognostic factor in AML [16].

The significantly inferior OS in patients remaining MRD positive at the time of CR (HR 2.44) highlights the prognostic utility of measurable residual disease. This mirrors findings from VIALE-A follow-up analyses, where achieving MRD negativity was a key predictor for extended survival [2, 17].

The viability of shortened venetoclax durations has practical consequences for patient management. Reducing the duration from 28 to 14 days may theoretically reduce venetoclax exposure and associated drug costs [3]. Additionally, shorter cycles may improve drug compliance and mitigate potential drug-drug interactions in elderly populations with multiple comorbidities [11].

This study has several limitations. The cohort was too small to definitively demonstrate that shortened venetoclax durations are non‑inferior to the standard 28‑day regimen. Because our cohort was from a single institution, retrospective, and non-randomized, patient characteristics differed across the venetoclax duration groups, and physicians’ treatment‑duration preferences may have influenced group assignment, introducing potential selection bias and limiting generalizability. We highlight common adverse events but have limited information on venetoclax dose modifications, cycle delays, cumulative dose, and reasons for discontinuation. As such, administration days may not fully capture actual drug exposure, and the observed cycle 1 dose difference may in part reflect concomitant CYP3A inhibitor use rather than differences in treatment intensity. While not reaching statistical significance, there was trend toward a higher prevalence of adverse ELN 2022 criteria and secondary AML in the 14-day cohort relative to the 21-day and 28-day groups. This together with the modest sample size, suggests a true imbalance in baseline characteristics between cohorts cannot be excluded. As adverse ELN 2022 risk and secondary AML are predictors of worse outcomes, their potential over-representation in the 14-day cohort would be expected to bias survival outcomes against this group.

In conclusion, this study did not detect a significant independent effect of shortened 14 or 21-day venetoclax cycles on outcomes for selected patients with newly diagnosed AML. Further data from the recently reported OPTI-AML trial (NCT03013998) and prospective data from the planned SEVENAZA study (NCT07082452), comparing HMA + 7-day against HMA + 28-day VEN, are necessary to establish a venetoclax dosing strategy.

## Supplementary Information

Below is the link to the electronic supplementary material.

Supplementary Material 1.

## Data Availability

The authors confirm that the data supporting the findings of this study are available within the article. Additional data are available on request from the corresponding author.
