# Supplementary material for: Optimizing venetoclax duration with hypomethylating agents for newly diagnosed acute myeloid leukemia: impact on response and survival
Source: Ann Hematol. 2026 Aug 4;105(10):415. doi: 10.1007/s00277-026-07219-2 (PMC13574922; doi:10.1007/s00277-026-07219-2)
Supplement: Supplementary file 1 [file 277_2026_7219_MOESM1_ESM.docx]

Figure S1: Oncoprint highlighting genetic alterations among our cohort.

Supplementary Table 1: CR/CRi rate stratified by ELN risk stratification.

| **Characteristic** | **14-day***^1^* | | **21-day***^1^* | **28-day***^1^* | **p-value***^2^* |
| --- | --- | --- | --- | --- | --- |
| **ELN 2024** | |  |  |  |  |
| Adverse | 4/5 (80%) | | 5/11 (45%) | 4/12 (33%) | 0.224 |
| Favorable | 5/12 (42%) | | 22/29 (76%) | 10/16 (63%) | 0.131 |
| Intermediate | 1/2 (50%) | | 4/7 (57%) | 3/3 (100%) | 0.533 |
| **ELN 2022** | |  |  |  |  |
| Favorable | 0/0 (NA%) | | 8/9 (89%) | 5/7 (71%) | 0.550 |
| Intermediate | 1/2 (50%) | | 8/10 (80%) | 2/5 (40%) | 0.268 |
| Adverse | 10/20 (50%) | | 15/28 (54%) | 10/19 (53%) | >0.999 |
| *^1^* n/N (%) | |  |  |  |  |
| *^2^* Fisher’s exact test | |  |  |  |  |

Supplementary Table 2: Multivariate models for OS, DFS, and CIR.

| **Characteristic** | **OS** | | | **DFS** | | | **CIR** | | |
| --- | --- | --- | --- | --- | --- | --- | --- | --- | --- |
|  | **HR***^1^* | **95% CI***^1^* | **p-value** | **HR***^1^* | **95% CI***^1^* | **p-value** | **HR***^1^* | **95% CI***^1^* | **p-value** |
| **ELN 2024 - Multivariate Models** | | | | | | | | | |
| **Age** | 0.98 | 0.96, 1.01 | 0.128 |  |  |  |  |  |  |
| **VEN duration** |  |  |  |  |  |  |  |  |  |
| 28-day | — | — |  | — | — |  | — | — |  |
| 21-day | 0.67 | 0.34, 1.32 | 0.246 | 0.79 | 0.37, 1.69 | 0.543 | 0.80 | 0.34, 1.89 | 0.620 |
| 14-day | 1.24 | 0.58, 2.66 | 0.582 | 1.38 | 0.54, 3.56 | 0.502 | 0.92 | 0.29, 2.90 | 0.890 |
| **ELN 2024** |  |  |  |  |  |  |  |  |  |
| Favorable | — | — |  | — | — |  | — | — |  |
| Adverse | 2.57 | 1.30, 5.08 | 0.007 | 2.43 | 1.13, 5.23 | 0.023 | 2.02 | 0.82, 4.95 | 0.120 |
| Intermediate | 1.79 | 0.69, 4.62 | 0.229 | 1.29 | 0.46, 3.60 | 0.626 | 1.11 | 0.29, 4.29 | 0.880 |
| **AML type** |  |  |  |  |  |  |  |  |  |
| De Novo | — | — |  | — | — |  | — | — |  |
| Secondary | 1.53 | 0.81, 2.88 | 0.191 | 1.06 | 0.50, 2.26 | 0.885 | 1.25 | 0.51, 3.07 | 0.620 |
| **ELN 2022 - Multivariate Models** | | | | | | | | | |
| **Age** | 0.99 | 0.97, 1.01 | 0.170 |  |  |  |  |  |  |
| **VEN duration** |  |  |  |  |  |  |  |  |  |
| 28-day | — | — |  | — | — |  | — | — |  |
| 21-day | 0.60 | 0.31, 1.16 | 0.130 | 0.76 | 0.37, 1.56 | 0.454 | 0.73 | 0.34, 1.58 | 0.420 |
| 14-day | 0.91 | 0.43, 1.93 | 0.798 | 1.02 | 0.40, 2.61 | 0.960 | 0.82 | 0.26, 2.59 | 0.740 |
| **ELN 2022** |  |  |  |  |  |  |  |  |  |
| Favorable | — | — |  | — | — |  | — | — |  |
| Intermediate | 1.90 | 0.62, 5.82 | 0.259 | 1.55 | 0.53, 4.56 | 0.427 | 0.72 | 0.20, 2.58 | 0.610 |
| Adverse | 2.60 | 0.98, 6.93 | 0.055 | 2.05 | 0.81, 5.23 | 0.132 | 1.27 | 0.46, 3.57 | 0.640 |
| *^1^* HR = Hazard Ratio, CI = Confidence Interval | | | | | | | | | |
